# Giant Coronary Sinus Complicated by Spontaneous Thrombosis

**DOI:** 10.14797/mdcvj.1159

**Published:** 2022-09-06

**Authors:** Otito Ojukwu, Salma Zook, Neal Kleiman, Gerald Lawrie, Mahwash Kassi

**Affiliations:** 1 Texas A&M School of Medicine, College Station, Texas, US; 2 Houston Methodist DeBakey Heart & Vascular Center, Methodist J.C. Walter Jr Transplant Center, Houston Methodist Hospital, Houston, Texas, US

**Keywords:** coronary sinus thrombosis, mitral regurgitation, echocardiogram, fistula, unroofed coronary sinus

## Abstract

Spontaneous coronary sinus thrombosis (CST) is an extremely rare occurrence. Most cases are iatrogenic and related to right heart instrumentation, due to either central line placement or electrophysiology procedures such as pacemaker insertion that causes direct damage to the endothelial lining. The course can be insidious and may result in a fatal outcome. Diagnosis of CST is challenging, and the syndrome often goes unrecognized. However, in the current era of multimodality imaging, it is possible that this condition will be recognized in more patients. Herein, we present a patient with spontaneous coronary sinus thrombosis that was diagnosed using multimodality imaging and thereafter successfully managed.

A 67-year-old retired male pilot previously in good health presented to the hospital with shortness of breath and chest pain. His past medical history was significant for hypertension and hyperlipidemia. Cardiac catheterization 15 years prior for an abnormal stress test revealed an arteriovenous (AV) fistula in the left circumflex coronary artery, which drained into the coronary sinus. But it showed no atherosclerotic disease.

On his current presentation, the patient had New York Heart Association class III symptoms and typical chest pain that radiated from his arm to his jaw. The patient was in atrial fibrillation and was suspected to have non-ST elevation myocardial infarction. Coronary angiography revealed a large aneurysm in the ostial left circumflex artery, beyond which the artery was completely occluded. The left anterior descending and right coronary artery both had minimal disease (Figure 1). No intervention was performed, but further imaging was pursued to better delineate the left circumflex aneurysm.

**Figure 1:**
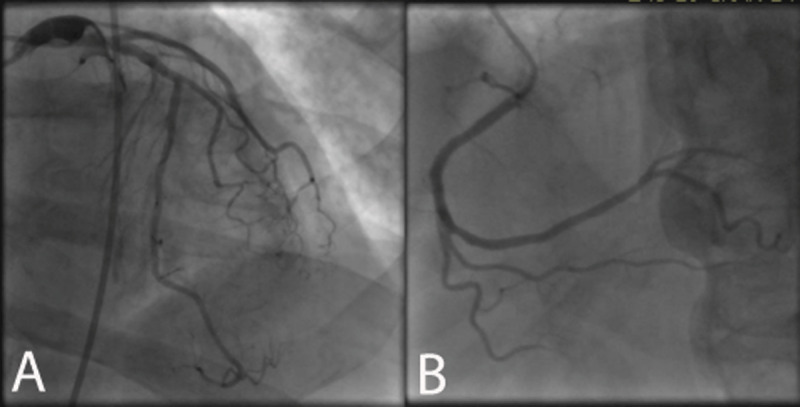
Coronary angiography revealed aneurysmal left circumflex artery with no significant disease in the left anterior descending or right coronary artery.

Echocardiography showed preserved ejection fraction (60-64%) but with inferior wall hypokinesis and significant mitral regurgitation. An unusual cystic structure was visualized posterior and lateral to the left atrium (Figure 2 A). Contrast injection did not reveal venous or arterial communication. Transesophageal echocardiogram revealed severe mitral regurgitation secondary to a tethered posterior mitral leaflet. The study suggested features of an unroofed coronary sinus atrial septal defect but was not conclusive due to significant clot burden (Figures 2 B, C; Video 1). Thereafter, further imaging was proposed.

**Figure 2:**
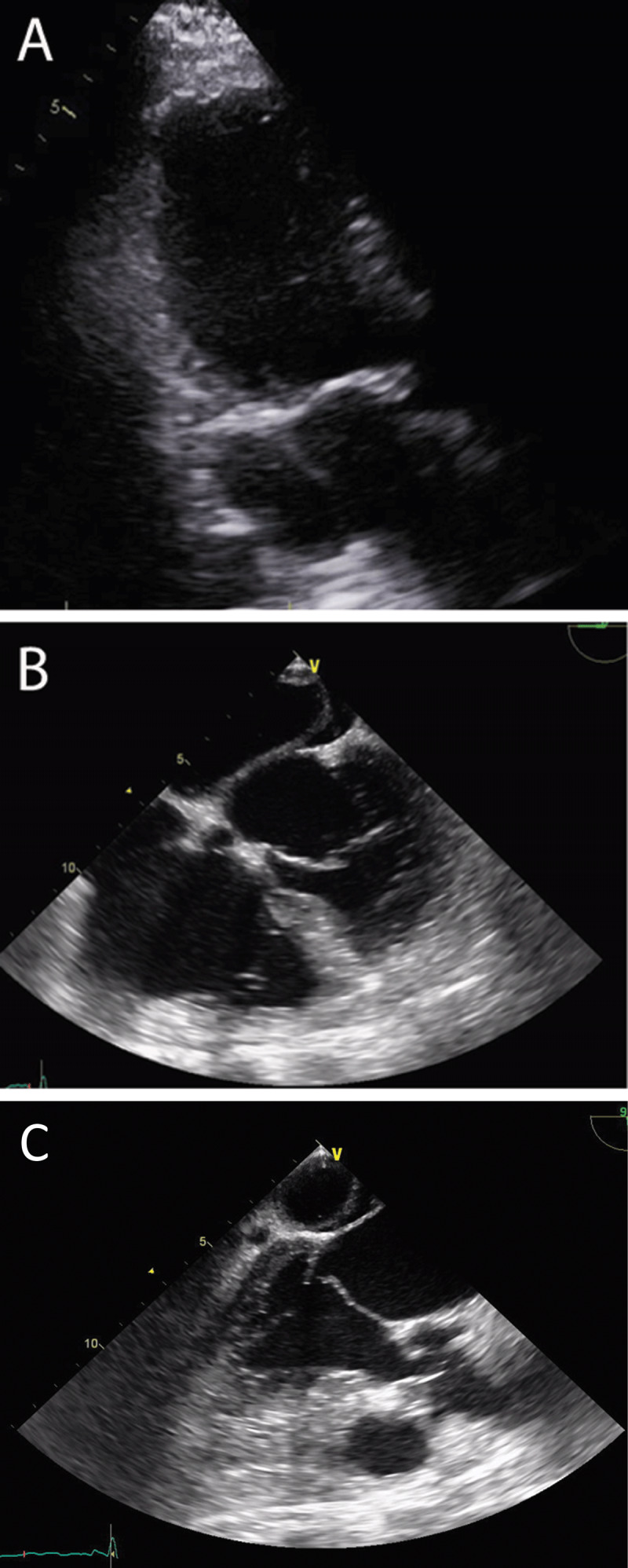
**(A)** Three-chamber view on transthoracic echocardiogram shows the cystic structure with no contrast flow after normal saline injection. **(B)** Four-chamber view on transesophageal echocardiogram showing the cystic structure in relation to the left atrium. **(C)** Tethered posterior leaflet.

**Video 1:** Transesophageal echocardiogram with color Doppler showing severe mitral regurgitation. see also at https://youtu.be/CDsc0Bmqym0

Cardiac magnetic resonance imaging showed a subacute transmural left circumflex infarction. The posterior mitral leaflet was fixed, and severe posteriorly directed mitral regurgitation (regurgitant volume 52 mL, regurgitant fraction 59%) and moderate tricuspid regurgitation were present. There was a large thrombus in the coronary sinus with complete obstruction of flow, resulting in significant dilation, 3.7 cm in maximal diameter. Cardiac computed tomography (CT) was performed to see if this was related to the known AV fistula that the patient had several years prior. The cardiac CT showed a nondominant left circumflex that was aneurysmal and thrombosed along its entire 12 mm course. There was a 6-cm by 4-cm aneurysmal and thrombosed coronary sinus. The CT did not demonstrate unroofed coronary sinus.

The patient thereafter had surgical mitral valve repair and was noted to have a ruptured tip of the posteromedial papillary muscle below the attachment of the chordae. A large thrombosed coronary sinus and aneurysmal left circumflex were visualized (Figure 3). The venous drainage of the heart was normal although there were variceal veins on the epicardium representing collateral venous flow. Absence of unroofed coronary sinus was confirmed on direct surgical visualization. The patient underwent mitral valve repair with insertion of four GORE-TEX® chordae (Gore Medical) to P3 segment and full ring annuloplasty with a 29-mm St Jude Attune ring (St Jude Medical). The postoperative course was uneventful.

**Figure 3:**
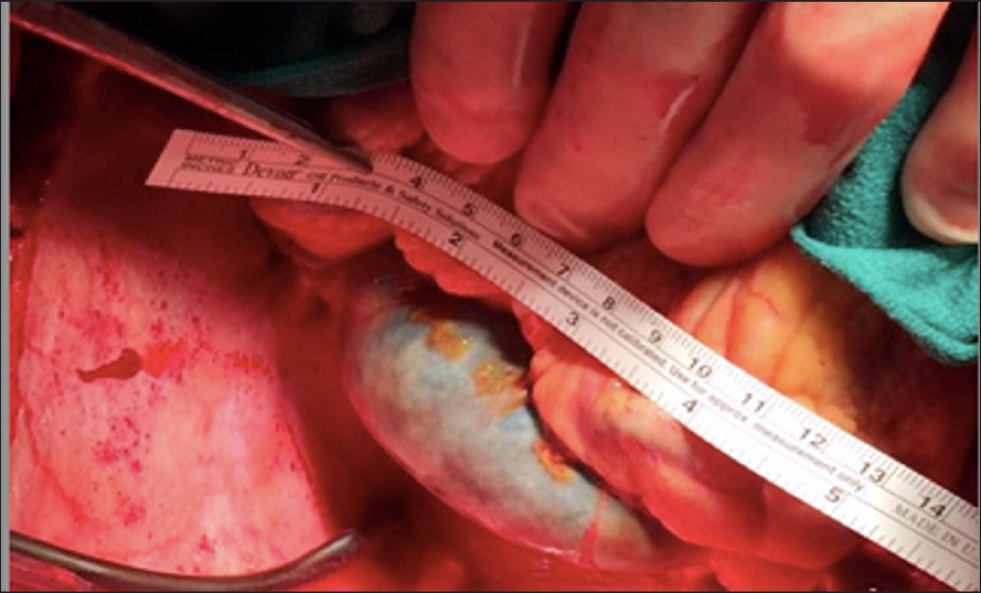
Surgeon’s view of thrombosed coronary sinuses.

## Points to Remember

- The coronary sinus receives about 60% of the cardiac venous return and empties into the right atrium. Coronary sinus thrombosis (CST) occurs when there is thrombus formation in the coronary sinus that could be secondary to obstruction, hypercoagulability, or direct endothelial injury.^1^^2^
- The mechanism by which thrombosis occurs is similar to how thrombus forms in other areas of the body. Virchow’s triad describes three mechanisms that contribute to thrombosis: endothelial damage, hypercoagulability, and venous stasis. These factors also apply to the development of CST.^2^
- If a dilated coronary sinus is visualized on imaging, it is prudent to consider underlying etiologies that caused dilation and ensuing thrombosis of the coronary sinus.^3^
- Once iatrogenic causes are ruled out, other etiologies to consider include congenital defects such as unroofed coronary sinus or presence of a fistula, which was the case in our patient.^4^ Valvular lesions, such as mitral or tricuspid regurgitation or coronary artery occlusion, may also result in development of CST. Some cases have been reported showing association with atrial fibrillation, hypercoagulability states, or even diseases such as Crohn’s disease.^1^
- Presentation of CST can be rather insidious where collateral flow in the venous system over years may cause minimal symptoms. However, there have been cases with more devastating presentation such as myocardial infarction, shortness of breath, cardiac tamponade, and even death.^1^ The severity of symptoms may depend on how acutely the thrombus formation occurred.
- Diagnosis is usually made with transesophageal echocardiogram, which also allows accurate representation of the coronary sinus. It is prudent to exclude the presence of an unroofed coronary sinus, which is an important consideration given that management may change.^4^
- In our case, due to heavy burden of thrombus, a multimodality imaging approach was more fruitful, and cardiac CT was particularly useful as it allowed for concomitant visualization of the coronary arteries.^3^
- Unroofed coronary sinus atrial septal defect is characterized by interatrial shunting between the coronary sinus and left atrium. It primarily occurs due to a cardiac congenital anomaly.^4^ If unroofed coronary sinus is the culprit, management may change because surgical correction is required. In patients with other valvular complications, valve repair may be necessary.^5^
- Due to complexity of disease, treatment varies case by case.^6^ Early detection is critical for successful outcomes in spontaneous CST and may be possible in the current era of multimodality imaging.
- Treatment with anticoagulant therapy has proven to be useful and may be considered for chronic CST.^1^
